# Implementation of Quality Indicators of Perinatal/Neonatal Palliative Care One-Year Following Formal Training

**DOI:** 10.3389/fped.2021.752971

**Published:** 2021-12-01

**Authors:** Charlotte Wool, Elvira Parravicini

**Affiliations:** ^1^Department of Nursing, College of Nursing and Health Professions, York College of Pennsylvania, York, PA, United States; ^2^Division of Neonatology, Department of Pediatrics, Columbia University Irving Medical Center, New York, NY, United States

**Keywords:** quality indicators, perinatal palliative care, neonatal palliative care, medical education, learning transfer

## Abstract

**Objective:** The aim of this study was to measure implementation of quality indicators (QIs) of Perinatal/Neonatal Palliative Care (PNPC) as reported by participants following a one-year training course.

**Study Design:** A cross-sectional survey mixed-method design was used to obtain data from an interdisciplinary team of professionals one year after attending a PNPC training course. A questionnaire with 32 QIs queried participants about self-reported implementation of PNPC and that of their colleagues. Descriptive and frequency data were analyzed to measure the implementation of PNPC QIs. Qualitative data were examined using content analysis.

**Results:** Response rate was 34 of 76 (44.7%). Half of the QIs are implemented in clinical settings by course attendees more than 90% of the time, and 15 QIs are implemented between 70 and 89.9%. Colleagues within the same healthcare system applied palliative care practices less frequently than those who attended the training course. When asked if quality indicators were “always” implemented by colleagues, the average difference in scores was 36% lower. Qualitative analyses resulted in three themes that addressed changes in clinical practice, and four themes that summarized barriers in practice.

**Conclusion:** There is high frequency of implementation of QIs by professionals who attended an evidence based PNPC training course. PNPC is implemented by the colleagues of attendees, but with less frequency. Attending evidence-based education increases clinicians' opportunities to translate quality PNPC care into clinical settings.

## Introduction

Perinatal/neonatal palliative care (PNPC) has evolved in the previous decades and is defined as a coordinated and comprehensive array of medical, nursing and supportive services provided to women who continue a pregnancy affected by a life-limiting fetal condition (LLFC). Several national organizations have endorsed PNPC and state that the obstetric and newborn care be centered on maximizing the quality of life and comfort for newborns (1). Aspects of PNPC should include prenatal consultation, development of a plan for birth, access to neonatal and pediatric specialists as needed, and full support, including bereavement care, during the pregnancy trajectory and postnatal period (1).

The National Consensus Project's (NCP) Clinical Practice Guidelines for Quality Palliative Care incorporates language that addresses perinatal palliative care and recommends that professionals in direct care of seriously ill patients should have both the training and experience to complete palliative assessments and address common elements reflective of the goals of quality palliative care (2). The NCP document provides structure for the essential elements of PNPC through eight domains. Each domain provides specific goals for achieving quality outcomes and acts as a framework by which the unique attributes of PNPC can be integrated. Quality indicators (QIs) are explicitly defined measurable items that refer to the processes, outcomes or structure of care provided to patients (3).

Formal training and education for PNPC is evolving but is not widely available despite recommendations from the AAP that it be included in all pediatric education, training curricula, and quality improvement (4). In response to the educational needs, Columbia University Irving Medical Center (CUIMC) developed a 3-day intensive training course to address the quality care and essential elements of PNPC. Attendees provided feedback on their self-reported competence directly before and after the course (5). This study builds upon the first to measure the implementation of core palliative care principles into the work environments of those who participated in the training and their colleagues.

This mixed-methods study had three specific aims. Aim #1 was to measure, by self-report, the implementation of PNPC QIs by course attendees. Aim #2 measured if professionals within the same health care system as the attendee implement PNPC within their health care system as reported by the attendee. Here we seek to understand if information from a training event extends among providers in the same clinical care environment. Aim #3, the qualitative portion of the study, explored self-reported changes in palliative care practice and barriers to its uptake.

## Methods

### Training Course

A three-day course entitled *The Next-Level Perinatal/Neonatal Comfort Care Training Course: Developing a Medical and Interdisciplinary Plan for Each Baby and Support for Their Family* was offered at CUIMC in June 2019. Coursework focused on the evidence base of PNPC through lectures, role-play, discussions, parent interviews, and hands-on demonstrations. The essential elements of PNPC and objectives of the course were intentionally organized into the eight domains from the NCP (2) so that quality indicators could be assessed and measured. Details about the training course were previously reported (5).

### Study Design

For this mixed method study, a cross-sectional survey was developed to gather demographic data and information about the implementation of interventions pertinent to PNPC one year following the training. Measurements were completed to examine: (1) 32 QIs outcome variables from within the NCP domains, and (2) two open-ended qualitative items as follows: “*Since attending the three-day training course, the three most significant changes in my palliative care practice have been…”* and “*Since attending the three-day training course, the three most significant barriers in palliative are are.”* Participants were provided informed consent at the outset of the survey. They were asked to rate if, and to what extent, they personally implemented the PNPC by responding on a forced Likert scale “always—sometimes—never.” Participants were then asked to what extent, if any, their professional colleagues implemented the PNPC interventions on a similar forced Likert scale that included “always—sometimes—never—unknown” as options. The survey was open for 12 weeks and participants were invited to complete the survey through three email reminders. Approval was obtained from the Columbia University Institutional Review Board (IRB-AAAS4060) and informed consent was obtained from participants at the start of data collection.

Participants with English as a second language and living outside the US were offered a meeting with course faculty to answer questions about the survey and discuss changes in their practice. This meeting was coordinated and then delivered via Zoom telephone conferencing, and lasted one hour.

### Analysis

Quantitative analyses for Aims #1 and #2 followed the same process. Step One: the 32 QIs were measured using frequency analyses. Aim #1 focused on responses labeled “always” and “sometimes” for attendees. Aim #2 examined how course participants rated their organizational colleagues' uptake of PNPC QIs. Step Two: For each QI, a total score of “always” plus “sometimes” was computed. These total scores were divided into the following thresholds: ≥ to 90, 80–89.9, 70–79.9 and <70%. Step Three: Means were calculated for the 32 QIs in the responses “always” and “sometimes.” Step Four: Comparative analyses was conducted to examine the means between the attendees and colleagues.

**Figure 1:**
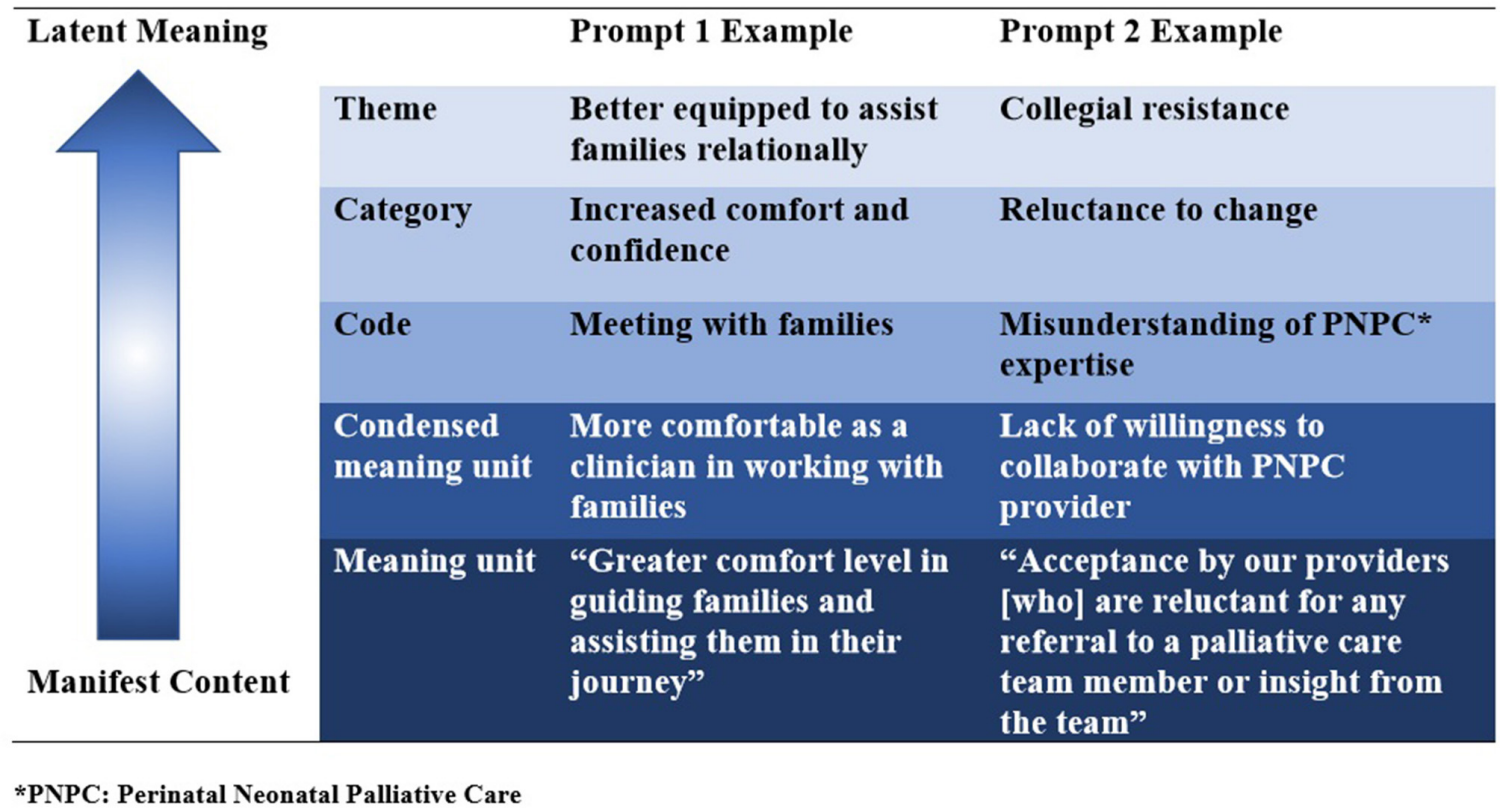
Examples of content analysis process.

For Aim #3, Krippendorf's content analysis was used to examine qualitative data. Content analysis is context sensitive, allowing the researcher to process data texts that are significant, meaningful, informative and representational to others (6). A strength of content analysis is the opportunity to increase our understanding of phenomena, in this case experiences of clinicians who attended a three-day training course and returned to their organization to operationalize what they learned. A systematic approach was used in which Author #1 (CW) mapped patterns of co-occurring words to identify clusters of common meanings, called units. The units were coded and placed in categories, and finally into themes. These steps were followed by a meeting of both authors in which results were reviewed and corroborated (Figure 1).

## Results

### Demographics

The web-based survey was administered to the 76 individuals who attended the three-day training course, 34 of whom responded to the entire survey, for a response rate of 44.7%. Most participants practiced in the United States (67%). Respondents were physicians (43.4%), nurses (28.2%) and other (28.2%) practicing in neonatology (63%), obstetrics (13%), palliative care (15%), and other (8.7%). The majority of respondents worked in an academic medical center (65%), and the remainder at a large regional hospital (17.4%), small community hospital (4.3%) and other (13%).

### Specific Aim #1: Self-Reported Implementation of QIs

**Figure 2:**
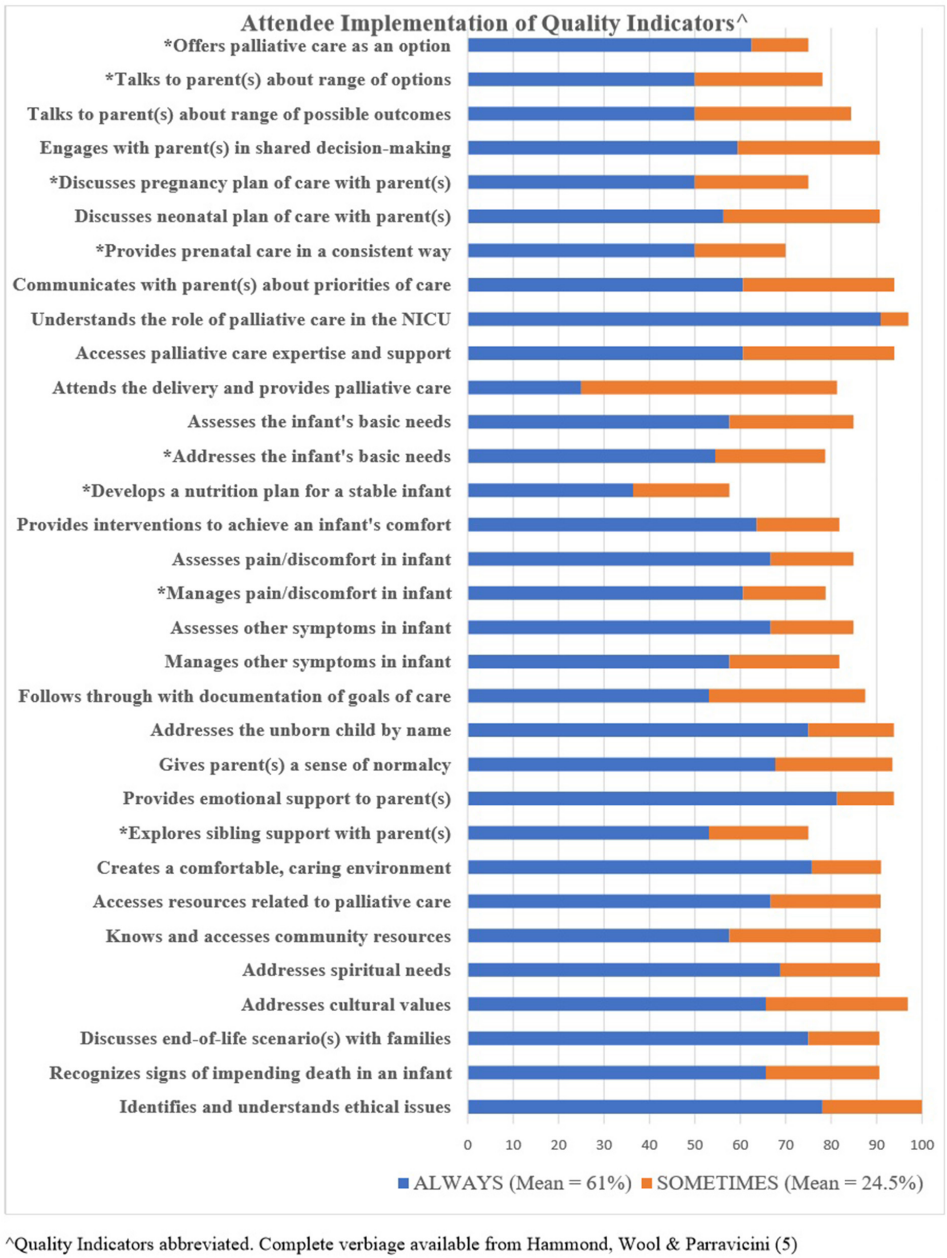
Attendee implementation of quality indicators.

**Figure 3:**
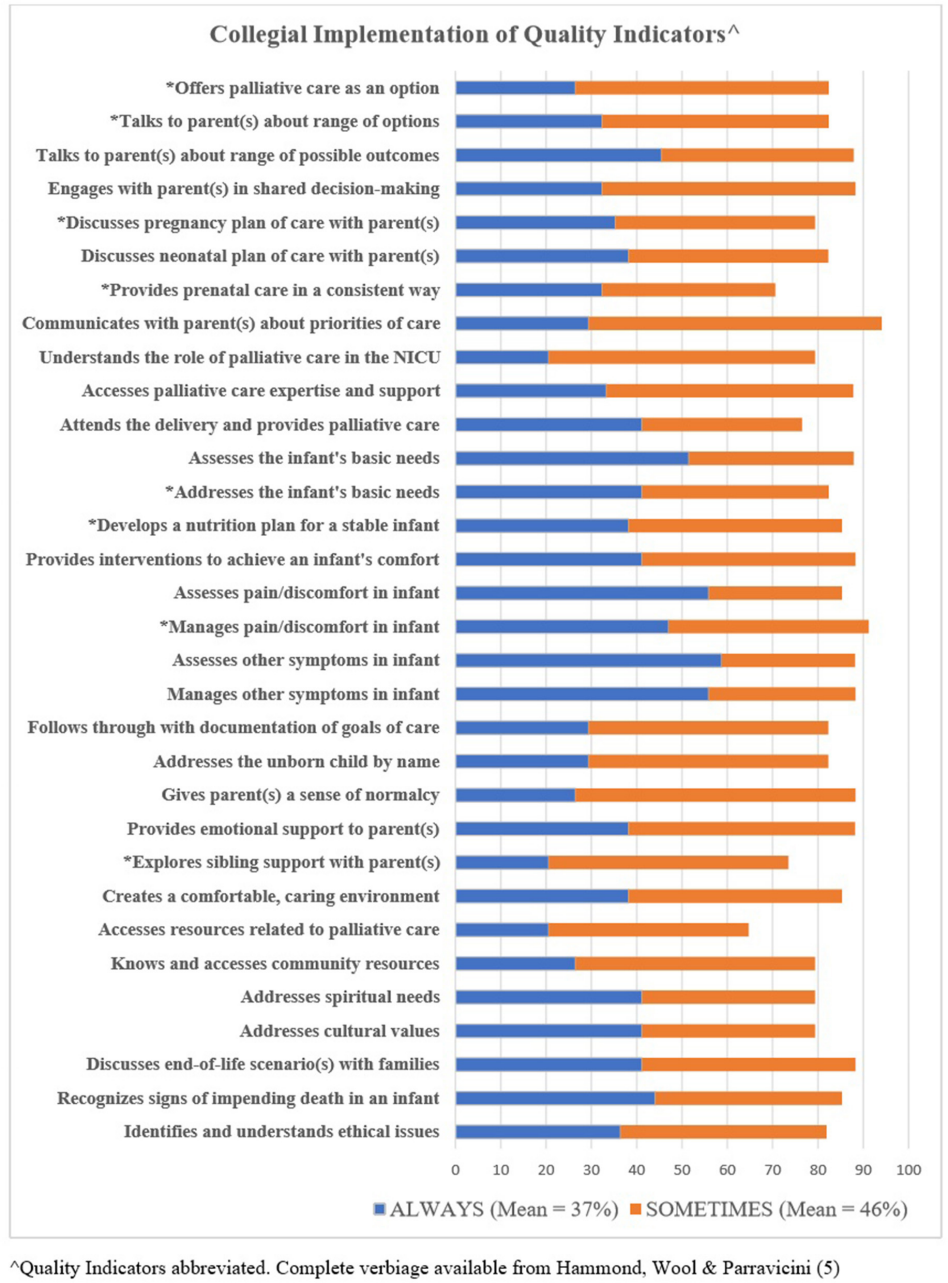
Collegial implementation of quality indicators.

Frequency data from attendees' self-reported responses for “always” and “sometimes” for each QI is shown in Figure 2. When each QI total score was computed, 16 of the QIs were in the highest threshold of ≥ to 90%, 8 were in the 80–89.9% threshold, and 7 were in the 70–79.9% threshold. One QI, developing a nutrition plan for an infant with a life-limiting condition who is breathing and stable on room air, was below the 70th percentage threshold. All QIs below the 79.9% threshold are indicated with an asterisk on Figure 3. The mean average of total scores indicating an “always” response was 61.3%, and those indicating a “sometimes” response was 24.5%.

### Specific Aim #2: Colleague Implementation of QIs as Reported by Course Attendees

Course attendees provided a report regarding collegial implementation for each QI. Figure 3 reflects responses for “always” plus “sometimes.” When each QI total score was computed, two of the QIs were in the highest threshold of ≥ to 90%, 21 were in the 80–89.9% threshold, and eight were in the 70–79.9% threshold. One QI, accesses resources related to perinatal palliative care (clinical resource materials, continuing education), was below the 70th percentage threshold. All QIs below the 79.9% threshold are indicated with an asterisk on Figure 3. The mean average of total scores indicating an “always” response was 37%, and those indicating a “sometimes” response was 46%.

### Specific Aim #3: Qualitative Results

The qualitative narrative included details of specific implementations that were a direct result of information provided within the training course. Ten participants reported at least one of the following: establishment of PNPC guidelines and policies in their institutions, commencement of a new PNPC service line, identification of champions to assist with programmatic processes, organization of formal PNPC teaching and training courses, and development of parents support groups.

Three themes, (1) better equipped to assist families, (2) expansion of interdisciplinary collaboration, and (3) improvements in hands-on expertise arose from the prompt “*Since attending the three-day training course, the three most significant changes in my palliative care practice have been:.”* Clinicians reported being “more equipped to handle these situations emotionally,” and five respondents specifically stated they were “more comfortable” relating to and assisting families, while six participants stated they had increased in “knowledge” about PNPC and felt more “empowered.” The second theme addressed positive changes in teamwork, where information about PNPC was being disseminated and team members had improved collaboration. Coordinated and collaborative efforts were mentioned in the context of multidisciplinary and interdisciplinary team efforts. The third theme focused on changes in skill sets. This theme included very specific details about translating information from the training course to patient care environments. For example, how to feed a baby, improve and standardize memory making, allocate resources, and change the physical environments to better serve families were coded into this theme. Changes in how to communicate PNPC to others were also noted, with one participant reporting “teaching others that palliative care is not about “doing nothing”.”

Four themes, (1) collegial resistance, (2) lack of resources, (3) improving skills, and (4) fragmentation of care arose from the prompt “*Since attending the three-day training course, the three most significant barriers in palliative care practice are:.”* Collegial resistance was noted as a barrier and respondents reported a lack of “buy in from obstetrics,” difficulty breaking the status quo within nursing protocols, and team members in positions of authority who are hesitant to change. The second theme targeted a general lack of resources, such as time, support, dedicated space to provide clinical care, funding, and adequately trained personnel. In the third theme, participants acknowledged the need to continue to increase PNPC skills such as creating molds for bereaved parents, managing pain and symptoms of the neonate, and communicating the importance and meaning of PNPC to colleagues. The fourth theme arose from codes that identified difficulties with referral pathways, inconsistencies in perinatal consult(s), and communication breakdowns among specialties. Three respondents stated Covid-19 was a barrier in their PNPC practice but did not elaborate. These responses were not coded or placed into a theme.

## Discussion

### Aim #1 and Aim #2 (Quantitative Findings)

This study demonstrates that translation of quality indicators into the clinical setting is associated with education provided in an evidence-based curriculum by an interdisciplinary team of experts. This result is in line with the NCP recommendation of reinforcing guidelines to develop, test, and implement quality indicators to work toward continual improvement of the quality of care (2).

Translational research is a gold standard and represents true transdisciplinary outcomes. It is characterized as harnessing the use of discoveries from basic science by applying the research findings into practice to improve care for patients (7). In all 32 QIs, attendees self-reported high percentages of QI implementation, and in 50% of the QIs, total rates surpassed the 90th percentile. The one QI exception to implementation was the development of a nutrition plan for a stable infant. This result is not unexpected, given that in the survey obtained at the time of the training course (5), participants reported the lowest competency score for this same item. Moreover, neonates with a life-limiting condition have a short life expectancy and most are terminally ill, thus nutrition is considered, but a formal plan for nutrition is not developed. While uncommon in infants nearing the end-of-life, hunger and thirst must be addressed as a part of palliative care. Colostrum care and non-nutritive sucking are recommended by the AAP to alleviate and pain and discomfort in neonates (8, 9) and are a natural extension of comfort care.

Our study sought to understand if palliative care that occurs within a healthcare system was recognized and acknowledged by the course attendees. Participants reported detailed information on how QIs were applied by their organizational colleagues. While implementation of PNPC QIs was reported, it was at lower frequencies. Less colleagues apply QIs “always” when compared to course attendees. This can be explained by a variety of potential factors including a lack of education or expertise (5), discomfort with PNPC (10, 11) or any of the many barriers addressed in these findings and others (12). Only two QIs reached the highest threshold when totaled, (1) communicating with parents about priorities of care and (2) managing pain and discomfort. Access to clinical resources and continuing education scored below the threshold of 70% and may be due to the participants' awareness of system-level barriers.

### Aim #3—Qualitative Findings: Significant Changes

In the year following a PNPC education, attendees were actively implementing specific patient care practices into the clinical setting. The narratives demonstrate that at least one third of the respondents established significant clinical and educational changes in their institutions, noting improvements in their communication skills and improved confidence to teach their colleagues about PNPC.

The comprehensive training eased discomfort that clinicians have reported in previous studies (13) as they recognized that the knowledge gained provided them with more confidence and assurance to support families anticipating the loss of an infant. These findings are in alignment with previous research that demonstrates the alleviation of helplessness, distress, and discomfort following comprehensive training (14).

Our study showed an expansion of interdisciplinary collaboration following a training course. One of the primary quality drivers of palliative care programs is collaboration among interdisciplinary team members (2). In PNPC this is especially important as mothers must navigate through the pregnancy, delivery, and postnatal periods, all of which involve clinicians with different areas of expertise. Expansion of collaborative efforts and trust among providers has potential to reduce fragmentation in care and improve patient satisfaction (15).

### Qualitative Findings: Significant Barriers

Our findings confirm other palliative care literature that recognizes fragmentation and recommends creating pathways and system-level routines to support optimal patient supports (16–19). Limited resources are often cited as a barrier to change (20), and while resource allocation can sustainably impact new services lines, challenges remain. Resistance to change is one of several challenges to organizational transformation. A variety of factors may contribute to collegial uptake of new services, including leadership effectiveness, readiness for change, roles and competencies needed to ensure the success of sustainable change, and individual commitment to and participation with new initiatives (21). In addition, health care systems often house silos of specialties which may hamper opportunities to connect and communicate with peers and can foster fragmentation of care. The necessary skill sets, which stem from a wide variety of interdisciplinary specialties and must usher patients through pregnancy, birth, and postnatal time periods add to complexity and challenges.

This study has several strengths. The current investigation follows another survey (5) where data from a pre-test, post-test design indicated that the PNPC training increased participant self-confidence. This study allowed measurements of attendees' implementation in clinical practice one year after the training. A second strength includes the diversity of the participants. In contrast with previous work (14) where attendees included mainly nurses in the field of obstetrics, a high number of physicians and several different disciplines were represented. The international representation (1/3 of the participants) is another strength.

This study has some limitations. Due to the study design, a comparison of implementation in before and after data could not be measured. Thus, associative relationships are demonstrated, but not direct cause and effect. While the survey response rate was average for a web-based survey, there is concern for potential non-response rate bias, in that those who did not respond may be those who did not implement QIs. Additionally, the data given by course attendees about the frequency of QI implementation by colleagues provide some initial insights, but does not comprehensively address all colleagues within a health care system.

In conclusion, education provided in an evidence-based curriculum by an interdisciplinary team of experts was associated with translation of PNPC quality indicators into the clinical setting, and implementation of clinical and education changes. The study allowed identification of specific barriers to PNPC practice. As PNPC continues to grow, this research helps support the usefulness of education and its practical applicability in clinical settings. Attendees in such courses make positive strides in implementation of quality indicators. Continuation of training will enable clinicians to improve knowledge, confidence, and embed lasting change into care for families.

## Data Availability Statement

The data set is secure and confidential. Corresponding author will share information by request when applicable. Requests to access the datasets should be directed to cwool@ycp.edu.

## Ethics Statement

The studies involving human participants were reviewed and approved by Columbia University IRB. The patients/participants provided their written informed consent to participate in this study.

## Author Contributions

CW and EP developed the survey, analyzed the data, and wrote the manuscript. All authors contributed to the article and approved the submitted version.

## Conflict of Interest

The authors declare that the research was conducted in the absence of any commercial or financial relationships that could be construed as a potential conflict of interest.

## Publisher's Note

All claims expressed in this article are solely those of the authors and do not necessarily represent those of their affiliated organizations, or those of the publisher, the editors and the reviewers. Any product that may be evaluated in this article, or claim that may be made by its manufacturer, is not guaranteed or endorsed by the publisher.
